# Survey of dose area product received by patients undergoing common radiological examinations in four centers in Nigeria

**DOI:** 10.1120/jacmp.v13i4.3712

**Published:** 2012-07-05

**Authors:** Bidemi I. Akinlade, Idowu P. Farai, Akintunde A. Okunade

**Affiliations:** ^1^ Department of Radiotherapy College of Medicine, University of Ibadan, University College Hospital Ibadan Oyo State Nigeria; ^2^ Department of Physics University of Ibadan Oyo State Nigeria; ^3^ Department of Physics Obafemi Awolowo University Ile‐Ife Osun State Nigeria

**Keywords:** radiological examination, dose area product (DAP), anti‐scatter grid, exposure factors, radiation protection

## Abstract

Patient dosimetry studies in diagnostic radiology in Nigeria have been on measurement of entrance skin dose and effective dose. Another important and easy to measure radiation dose descriptor that could be used to assess patient dose in radiological procedures is dose area product (DAP). Knowledge of DAP with location and projection of X‐ray beam allows direct calculation of organ dose and effective dose. In this study, DAP for commonly performed radiological examinations (abdomen, chest, lumbo sacral joint, pelvis, paranasal sinus, and skull) in four diagnostic centers in Nigeria were determined. These centers comprise of three classes of health care center namely tertiary, private, and specialist hospitals. Mathematical method was used to determine DAP received by 336 patients undergoing radiological examinations at the selected diagnostic centers. The DAP received by patient from each radiological examination varies from center to center. The range factor (RF) of DAP for individual patients ranged from 1.55–4.56, while RF of DAP among the selected centers was 2.27–55.84. The highest RF of DAP (55.84) was obtained from X‐ray examination of the chest. This variation was due to the application of anti‐scatter grid, and high kVp and high mAs values for chest examination by only one of the centers. The very wide variation in DAP found among diagnostic centers in this survey showed that there is need to harmonize radiological techniques of common X‐ray examination among different diagnostic centers. This would ensure optimal protection of patient against excessive radiation dose.

PACS numbers: 87.53.Bn; 87.59.B

## I. INTRODUCTION

Dose area product (DAP) is a product of surface area of patient that is exposed to radiation at the skin entrance multiplied by the radiation dose at this surface. Measurement of dose area product is suitable for achieving optimum degree of safety during radiological examination of patient. DAP is a valuable radiation dose descriptor because radiation‐induced bioeffects are directly related to both the magnitude of the radiation dose and the total amount of tissue that is irradiated.^(^ ^1^ ^)^ Also, DAP is useful for continuous quality assurance, as well as analysis of performance of X‐ray machines.

Dose area product could be measured by two methods, namely: (i) direct measurement through the use of a transmission ionization chamber at the surface of the X‐ray tube collimator; and (ii) by mathematical approach (indirect). The mathematical approach involves the product of irradiated area of the patient and radiation dose incident at the surface. In conventional diagnostic procedure, the entrance surface dose is considered a useful first approach in measurement of radiation exposure to patients.^(^ ^2^ ^)^ This is because the amount of radiation dose delivered to the skin of the patient determines both the stochastic and deterministic risks. The measurement of entrance surface dose with thermoluminescence dosimeter (TLD) has been shown to be laborious, capital intensive, and potentially intrusive,^(^ ^3^ ^)^ especially when large numbers of patients are involved in the survey. This made dosimeter‐based entrance surface dose measurement in routine X‐ray examination quite expensive for centers with limited resources. The adoption of a mathematical method for dose determination provides an avenue for greater output with respect to patient dose information. A study by McParland^(^ ^3^ ^)^ has shown that entrance skin dose can be estimated from dose area product with an accuracy of 30%–40%. Apart from being useful for estimating entrance skin dose in routine X‐ray examination, knowledge of DAP, as well as location and projection of X‐ray beam, can also be used to estimate effective dose, a quantity mostly used to assess stochastic risk from nonhomogeneous irradiation.^(^ ^4^ ^)^ DAP is easier to measure than entrance skin dose (ESD) and effective dose, especially in routine X‐ray examinations. Among several indirect methods employed for practical estimation of effective dose in conventional radiology and fluoroscopy is dose area product.^(^ ^5^ ^,^ ^6^ ^)^

In Nigeria, most of the studies on patient dosimetry in routine X‐ray examinations are usually dosimeter‐based measurement of either ESD or effective dose.^(^ ^7^ ^,^ ^8^ ^,^ ^9^ ^)^ However, the dose area product received by patients during the examination procedure is not known. This present study is aimed at measuring dose area product received by patients undergoing common radiological examinations in some diagnostic centers in Nigeria. It is anticipated that the results from this study would provide useful means of estimating DAP and effective dose received by patients during routine X‐ray examination, thereby providing easy and timely patient dosimetry in diagnostic radiology. The results of the present study were compared with DAP values published in literatures for similar radiological examinations.

## II. MATERIALS AND METHODS

This study was carried out in four diagnostic centers in Nigeria. These centers were comprised of tertiary hospitals (T), private diagnostic center (P), and a specialist hospital for women and children (S). The centers include University College Hospital (UCH), Ibadan (T); Obafemi Awolowo University Teaching Hospital Complex (OAUTHC), Ile‐Ife (T); TwoTees diagnostic centre (TDC), Yemetu Ibadan (P); and National Hospital Abuja (NHA) (S).

These centers were chosen for the study because of the large workload of patients they recorded per day. Apart from this criterion, all the centers, except NHA, are referral centers for routine chest radiography for most health care centers in the South Western region of Nigeria. From each center, information about the X‐ray unit was obtained from the manufacturer's manual available at each center. This information includes manufacturer's name, model of the X‐ray unit, year of installation, film type/speed and tube filtration. A total number of 336 patients who consented to participate were enrolled in this study. The selection criterion was based on weight of the patient. This is because various authors have observed that patient size influences dosimetry results; for uniformity in sizes, it has been suggested that only patients whose weights are within the range of 60–80 kg should be included in a patient dose survey.^(^ ^10^ ^,^ ^11^ ^)^ Therefore, the patients considered in this study were limited to those whose weights are within the range 60–80 kg. The radiological examinations considered in this study were abdomen, chest, lumbar sacral joint (LSJ), paranasal sinus (PNS), pelvis, and skull. These X‐ray examinations are considered to be the most common X‐ray examination performed on patients worldwide.^(^ ^12^ ^)^ The transmission ionization chamber required for direct measurement of DAP was not available at any of the centers selected for this study; therefore, the mathematical method, which is the alternative option for centers without transmission ionization chamber, was used in this study. Some of the quantities required for calculation of DAP for each patient include tube loading (mAs), tube voltage (kVp), focus to skin distance (FSD), focus to film distance (FFD), collimator size (beam area), and beam output of X‐ray machine. The body mass index (BMI) of all patients was also calculated. The BMI, which was derived from $\text{weight}/{\left(\text{height}\right)}^{2}$, is a useful classification scheme for the size and shape of a person.^(^ ^13^ ^)^ In this present study, DAP was calculated using the following equation:^(^ ^6^ ^)^

(1) $$DAP(mGycm^{2})=L(mAs)Do(mGy/mAs)A{\left(cm^{2}\right)}_{\left(FSD\right)}$$

where, *L* is the tube loading expressed in mAs; $D_{o}$ is the normalized beam output in mGy/mAs at 1 m; *FSD* is the focus to skin distance, and $A_{\left(FSD\right)}$ is the cross‐sectional area of the beam on the skin of the patient. The beam output (mR/mAs) of X‐ray machine was measured in all the centers with noninvasive X‐ray test device model 4000 M+ (Nuclear Associates, Carle Place, NY). The calibration of the X‐ray test device issued by the manufacturer was still valid at the time of this study. The distance between the radiation source and the detector was initially set at 60 cm to prevent back scattering of radiation, before reaching the detector. The inverse square law relation ($I\alpha 1/d^{2}$) was used to determine the beam output (mR/mAs) at 100 cm. A conversion factor of 0.00873 was applied to convert the beam output from mR/mAs to mGy/mAs.^(^ ^14^ ^)^ The radiographic films used in all the centers selected for this study was Kodak (Eastman Kodak Company, Rochester, NY) with medium speed of 200. All data from patients and X‐ray machines were analyzed with SPSS 16.0 for windows.

## III. RESULTS

The beam output (μGy/mAs) of X‐ray machines measured at tube voltage 80 kVp at each center is presented in Table 1. Also presented in Table 1 are technical parameters of X‐ray machine such as X‐ray tube model, year of manufacture, total filtration, for all the centers. From the year of installation to the time of this study, the age (years) of X‐ray tube at UCH, OAUTHC, TDC, and NHA was 30, 23, 6, and 5, respectively. The demographic data of patients such as age, weight, height, sex distribution, and BMI by radiological examinations and diagnostic centers are presented in Table 2. The overall age range of the study sample was 18–90 years. The summary of the exposure parameters and radiographic techniques such as kVp, mAs, FSD, FFD, and anti‐scatter grid applied for various radiological examinations considered in this study at different centers are presented in Table 3. While the range of kVp selected at UCH, OAUTHC, TDC, and NHA for all examinations was 55–93, 100–110, 75–90, and 70–81, respectively, the range of mAs values was 9 –300, 80–200, 60–120, and 40–81, respectively. Table 4 shows the DAP obtained for different X‐ray examination at the centers and these are compared with DAPs reported in some literatures for similar X‐ray examinations. The range of DAP ($\text{mGy}\;{\text{cm}}^{2}$) obtained from UCH, OAUTHC, TDC, and NHA was 100–783, 1663–7584, 122–634, and 113–694, respectively. The range factor of DAPs for individual patients for each of the radiological examination considered in this study is presented in Table 5. Also presented in Table 5 is the range factor of DAPs among the selected centers. The range factors (RF) of DAP for individual patients was 1.55–4.56, while RF among centers was 2.27–55.84.

**Table 1:** Radiographic rechnical data of X‐ray machines.

*Centre*	*X‐ray Tube*	*Model*	*Yr. of Installation*	*Total Filtration (mmAI)*	*Beam Output at 80 kVp (* $\mu Gy\;mAs^{-1}$ *)*
*This Study:*					
UCH	Roentgen 501	CK 3415	1974	2.7	11.60
OAUTHC	Shimadzu	R‐20	1981	1.7	5.20
TDC	Roentgen 201	R‐3149	1998	2.7	4.90
NHA	Philips Optimus	98011519	1999	1.0+0.1 mmCu	12.10
*From Study in Sudan (Suliman et al., 2006):*					
OTH	Shimadzu	Radiotex	2004	3.3	48.2
KTH	Shimadzu	Radiotex	2004	2.5	66.1

**Table 2:** Patient information for selected examinations with median values and range (in parenthesis).

			*Centers Selected for this Study*		
*Examination*	*UCH*		*OAUTHC*	*TDC*	*NHA*
Abdomen	Projection	AP		AP, PA	PA
	Male	3		3	1
	Female	0		1	1
	Age (yr)	46 (31–64)		56 (54–58)	58 (58–58)
	Weight (kg)	62 (62–74)		73 (69–77)	80 (80–80)
	$\text{BMI}({\text{kgm}}^{-2})$	23 (23–25)		25 (23–26)	24 (24–24)
Chest	Projection	PA	PA	PA	PA, LAT
	Male	23	54	51	49
	Female	23	24	24	25
	Age (yr)	33 (18–82)	23 (18–85)	22 (18–90)	23 (18–60)
	Weight (kg)	67 (60–78)	64 (60–76)	65 (60–80)	64 (60–80)
	$\text{BMI}({\text{kgm}}^{-2})$	24 (23–26)	23 (23–26)	24 (23–26)	23 (22–26)
LSI	Projection	AP, LAT		AP, LAT	AP. LAT
	Male	4		1	1
	Female	12		2	2
	Age (yr)	48 (34–71)		50 (22–51)	40 (40–40)
	Weight (kg)	62 (60–75)		69 (63–69)	80 (80–80)
	$\text{BMI}({\text{kgm}}^{-2})$	24 (23–26)		23 (23–23)	24 (21–26)
Pelvis	Projection	AP, LAT		AP	AP
	Male	1		1	1
	Female	3		2	1
	Age (yr)	49 (30–57)		34 (22–60)	55 (50–60)
	Weight (kg)	71 (65–75)		67 (63–68)	63 (62–64)
	$\text{BMI}({\text{kgm}}^{-2})$	24 (23–25)		26 (23–26)	23 (22–23)
PNS	Projection	PA, LAT		PA	
	Male	8		1	
	Female	10			
	Age (yr)	31 (18–66)		22 (22–22)	
	Weight (kg)	69 (60–75)		68 (68–68)	
	$\text{BMI}({\text{kgm}}^{-2})$	24 (23–25)		23 (23–23)	
Skull	Projection	AP, LAT			AP, LAT
	Male	4			
	Female				2
	Age (yr)	47 (46–47)			46 (46–46)
	Weight (kg)	70 (62–77)			77 (77–77)
	$\text{BMI}({\text{kgm}}^{-2})$	25 (24–26)			21 (20–22)

Notes: LSI= lumbo sacral joint; PNS = paranasal sinus; AP = anteroposterior; LAT = lateral.

**Table 3:** Exposure parameters and radiographic technique for selected X‐ray examinations with median values and range (in parenthesis).

			*Centers Selected for this Study*		
*Examination*		*UCH*	*OAUTHC*	*TDC*	*NHA*
Abdomen	Projection	AP		AP, PA	PA
	Tube voltage (kVp)	80 (78–84)		80 (80–80)	81 (81–81)
	mAs	80 (60–90)		85 (60–120)	40 (40–40)
	FFD (cm)	90 (90–90)		90 (90–90)	100 (100–100)
	FSD (cm)	75 (72–75)		74 (70–76)	82 (82–82)
	Antiscatter grids	Yes		Yes	Yes
Chest	Projection	PA	PA	PA	PA, LAT
	Tube voltage (kVp)	65 (60–84)	110 (100–110)	80 (75–90)	73 (70–77)
	mAs	12 (9–15)	128 (90–200)	39 (39–75)	25 (20–32)
	FFD (cm)	150 (150–150)	150 (150–150)	150 (150–150)	150 (150–150)
	FSD (cm)	133 (126–138)	134 (126–138)	135 (123–139)	135 (126–138)
	Antiscatter grids	No	Yes	No	No
LSI	Projection	AP, LAT	NA	AP, LAT	AP, LAT
	Tube voltage (kVp)	86 (70–93)		80 (80–85)	79 (77–81)
	mAs	150 (9–300)		120 (75–150)	36 (32–40)
	FFD (cm)	90 (90–100)		90 (90–90)	100 (100–100)
	FSD (cm)	72 (64–85)		66 (58–76)	78 (74–82)
	Antiscatter grids	Yes		Yes	Yes
Pelvis	Projection	AP, LAT	NA	AP	AP
	Tube voltage (kVp)	80 (75–86)		80 (75–85)	76 (70–81)
	mAs	75 (45–120)		120 (60–120)	33 (25–40)
	FFD (cm)	90 (90–100)		90 (90–90)	100 (100–100)
	FSD (cm)	76 (73–83)		75 (73–77)	84 (81–87)
	Antiscatter grids	Yes		Yes	Yes
PNS	Projection	PA, LAT	NA	PA	NA
	Tube voltage (kVp)	70 (55–90)		80 (80–80)	
	mAs	53 (18–90)		120 (120–120)	
	FFD (cm)	90 (90–90)		90 (90–90)	
	FSD (cm)	78 (70–88)		68 (68–68)	
	Antiscatter grids	Yes		Yes	
Skull	Projection	AP, LAT	NA	NA	AP, LAT
	Tube voltage (kVp)	74 (65–80)			70 (66–73)
	mAs	53 (30–90)			18 (16–20)
	FFD (cm)	90 (90–90)			100 (100–100)
	FSD (cm)	76 (72–80)			89 (88–90)
	Antiscatter grids	Yes			Yes

Notes: LSI= lumbo sacral joint; PNS = paranasal sinus; AP = anteroposterior; PA = posteroanterior; LAT = lateral; NA = not available.

**Table 4:** Comparison between DAP (median values and range (in parenthesis)) obtained in the present study with published DAP (mean values) data.

		*DAP (* $mGy.cm^{2}$ *) per Radiological Examination*				
	*Abdomen AP, PA*	*Chest PA, LAT*	*LSJ AP, LAT*	*Pelvis AP, LAT*	*PNS PA, LAT*	*Skull AP, LAT*
*This study:*						
UCH						
Male	661 (539–719)	142 (104–209)	350 (230–575)	501	230 (109–488)	433 (228–783)
Female	NA	127 (104–187)	437 (137–992)	368 (163–574)	422 (100–522)	433 (228–783)
OAUTHC						
Male	NA	4853 (2026–7584)	NA	NA	NA	NA
Female	NA	3950 (1663–6173)	NA	NA	NA	NA
TDC						
Male	452 (507–634)	206 (156–373)	263 (193–333)	454 (274–634)	166 (122–211)	NA
Female	434 (317–551)	193 (168–206)	333 (199–416)	488 (295–681)	247 (211–283)	NA
NHA						
Male	694	258 (182–388)	NA	279	NA	153 (113–192)
Female	NA	303 (182–388)	585 (476–694)	694	NA	NA
*Other studies:*						
Theocharopoulos et al.(^6^)	700	170	NA	NA	NA	310
Nickoloff et al.(^1^)	3850	400	1900	NA	NA	NA
Hart et al.(^18^)	3000	120	3000	3000	NA	NA
Hart and Wall(^20^)	2600	110	2550	2100	NA	635

Notes: LSJ = lumbo sacral joint; PNS = paranasal sinus; AP = antero−posterior; PA = postero−anterior; LAT = lateral; NA = not available.

**Table 5:** Range factor of DAPs for individual patient and among the selected centers.

*Radiograph*	*Projection*	*Range Factor for Individual Patients*	*Range Factor of DAP Among Centers*
Abdomen	AP, PA	1.55	2.27
Chest	PA, LAT	2.62	55.84
LSJ	AP, LAT	4.54	10.11
Pelvis	AP, LAT	3.80	7.98
Skull	AP, LAT	2.54	6.93

Notes: LSJ = lumbo sacral joint; AP = antero−posterior; PA = postero−anterior; LAT = lateral.

## IV. DISCUSSION

Dose area product (DAP) is a good indicator of radiation risk to patient and not just absorbed dose received by patient during X‐ray examination. This is because DAP reflects not only radiation dose to patient, but also the area of tissue irradiated.^(^ ^15^ ^)^ DAP is a surrogate of measurement for the entire amount of energy delivered to patient by the radiation beam. Although most of the X‐ray machines considered in this study are aged, they are still providing clinical services to Nigerian patients. Hence, the need to measure DAP received by patients during the procedure and thereby assess the performance of these old (> 15 years) X‐ray machines.^(^ ^16^ ^)^

As presented in Table 1, most of the X‐ray machines considered in this study were manufactured in the nineteenth century (1972–1999), unlike the twentieth century (2003–2005) X‐ray machines reported on in the Sudan.^(^ ^17^ ^)^ Of all the X‐ray tubes considered in this study, only the unit at OAUTHC contained total filtration (1.7 mmAl) that is below the range of values (2.5–4.3 mmAl) recommended for a good radiological practice, as reported in the UK.^(^ ^18^ ^)^ The beam output of X‐ray machines considered in this study was found to be very low when compared with beam output of similar but recently manufactured X‐ray machines reported in the Sudan survey. The technological advancement involved in the recently manufactured X‐ray units could contribute to this variation in beam output. When compared with a beam output measured at similar kVp in one of the teaching hospitals (Omdurman Teaching Hospital (OTH)) in the Sudan, the percentage of the beam output measured at UCH, OAUTHC, TDC, and NHA was found to be 24%, 11%, 10%, and 25%, respectively, of the values obtained in the Sudan. It is important to note that all the X‐ray machines considered in the Sudan study were installed between 2003 and 2005.

The age range (18–90 years) of patients considered in this study is wider than the previous patient dose survey (40–85 years) conducted in Nigeria.^(^ ^7^ ^)^ However, the study sample is within age range considered in Malaysia (14–92 years),^(^ ^19^ ^)^ UK (16–99 years,^(^ ^18^ ^)^ and the Sudan (16–90 years).^(^ ^17^ ^)^ The weight limit of 60–80 kg (mean 70 kg) was used as a criterion for selection of patients. This weight range is adopted to eliminate patient size factor, which usually influence patient's dosimetry results. The BMI of the study sample was found to be in the range of 21–26. All the patients who satisfied the weight criterion at OAUTHC at the time of this study were those with chest X‐ray examination. In all the centers, chest X‐ray examination has the highest frequency of radiological examination. It is well known globally that chest radiography is common and is usually the most patronized examination. The present study sample has 204 (61%) males and 132 (39%) females.

In all the centers, Kodak films with medium speed of 200 were used. One would expect all the centers to select exact exposure parameter for similar X‐ray examination since the film speed is similar, but this is not the case. As seen in Table 3, the range of tube voltages, kVp (60–110 kVp) selected for most of the radiological examinations in this study were within the values selected in the UK (50–150 kVp) survey^(^ ^20^ ^,^ ^21^ ^)^ and Malaysia (55–125 kVp). In the case of tube loading (mAs) settings, the range of values selected for most projections in this study (9–300 mAs) were within the values reported in the UK survey (5–485 mAs) but higher than those selected in Malaysia (9–122 mAs). The variation in tube loading was due to patient's size. For instance, the patients considered in Malaysia were light weight (60 kg) as compared with those in this study (60–80 kg) and the UK (65–75 kg) surveys. The range of values of FFD (90–150 cm) and FSD (58–139 cm) obtained from this study were within the optimum values of FFD (80–210 cm) required for good geometric image sharpness reported in Sudan. The technique of applying anti‐scatter grids for a given type of X‐ray examination was similar in all the centers except for chest examination, where only one centre, OAUTHC, applied anti‐scatter grids.

In Table 4, a comparison between the DAPs obtained in this study with DAPs reported in some literatures for similar radiological examinations of adult patients is presented. However, the comparison with the UK National reference doses was reported in this study due to similarity in the size (70 kg) of patient considered. From Table 4, it can be seen that DAPs obtained from abdominal AP and PA examinations at UCH ($661\;\text{mGy}\;{\text{cm}}^{2}$), TDC ($451\;\text{mGy}\;{\text{cm}}^{2}$), and NHA ($694\;\text{mGy}\;{\text{cm}}^{2}$) diagnostic centers are within the UK National reference doses (2600–$3000\;\text{mGy}\;{\text{cm}}^{2}$). The lowest DAP ($109\;\text{mGy}\;{\text{cm}}^{2}$) obtained for chest PA and LAT examinations was observed at UCH, while the highest ($3970\;\text{mGy}\;{\text{cm}}^{2}$) was obtained at OAUTHC. The value obtained at UCH is within the UK National reference doses (110–$120\;\text{mGy}\;{\text{cm}}^{2}$), whereas the DAPs obtained from other centers — namely OAUTHC ($3970\;\text{mGy}\;{\text{cm}}^{2}$), TDC ($168\;\text{mGy}\;{\text{cm}}^{2}$), and NHA ($258\;\text{mGy}\;{\text{cm}}^{2}$) — are higher than the UK National reference doses. The use of high kVp (100–110) technique and high mAs values (80–200) at OAUTHC for chest X‐ray examination could be responsible for the very high DAP ($3970\;\text{mGy}\;{\text{cm}}^{2}$) obtained at the center, as compared with other three centers that did not apply anti‐scatter grids. The DAPs obtained for AP and LAT lumbo sacral joint (LSJ) examination at UCH ($378\;\text{mGy}\;{\text{cm}}^{2}$), TDC ($333\;\text{mGy}\;{\text{cm}}^{2}$), and NHA ($585\;\text{mGy}\;{\text{cm}}^{2}$) are within the UK National reference dose (2550–$3000\;\text{mGy}\;{\text{cm}}^{2}$). Similarly, the DAPs obtained for AP and LAT pelvis examination at UCH ($332\;\text{mGy}\;{\text{cm}}^{2}$), TDC ($634\;\text{mGy}\;{\text{cm}}^{2}$), and NHA ($486\;\text{mGy}\;{\text{cm}}^{2}$) are within the UK National reference dose (2100–$3000\;\text{mGy}\;{\text{cm}}^{2}$). The DAP obtained for PA and LAT paranasal sinus (PNS) examination at UCH (the only center that had patients for this examination during the period of the study) was $166\;\text{mGy}\;{\text{cm}}^{2}$. DAP for PNS examination was not reported in the UK National survey report. The DAPs obtained for AP and LAT skull examination at UCH ($433\;\text{mGy}\;{\text{cm}}^{2}$) and TDC ($153\;\text{mGy}\;{\text{cm}}^{2}$) are within the UK National reference dose ($635\;\text{mGy}\;{\text{cm}}^{2}$).

The range factor (RF) used in this study is the maximum‐to‐minimum ratios of DAP values obtained from each of the radiological examination for individual patients and among the selected centers. As seen in Table 5, the range factor of DAP for individual patient was found to vary from 1.55 to 4.54, whereas the range factor of DAP among diagnostic centers for similar X‐ray examination was found to vary from 2.27–55.84. This variation is due to different radiographic technique employed by each center for similar radiological examinations. This technique includes choice of exposure factors, focus to film distance, filtration in the tube, the use of anti‐scatter grid, and beam output of X‐ray machine used. The wide variation in values of DAP among centers for similar X‐ray examination suggested that significant reduction in DAP from these X‐ray examinations would be possible without adversely affecting image quality. For instance, in the X‐ray examination of chest, three (75%) out of the four centers did not apply anti‐scatter grid and to the best of our knowledge, the image quality satisfied the diagnostic information required by the referral doctors.

## V. CONCLUSIONS

The dose area product (DAP) received by patient from common radiological examinations (abdomen, chest, lumbo sacral joint (LSJ), pelvis, paranasal sinus (PNS), and skull) in four diagnostic (tertiary, private, and specialist hospital) centers in Nigeria have been determined using mathematical method. The calculated dose area product (median) received by patient from most of the X‐ray examinations per center are within the values (mean) reported in the UK National reference doses. The only exceptions were the DAP values received from chest X‐ray examination, which was higher at OAUTHC, TDC, and NHA. Among these three centers, the DAP obtained from chest X‐ray examination at OAUTHC was very high and the increase was traceable to the use of high kVp technique with high mAs values.

The very wide variation of DAP (2.27–55.84) obtained among the selected centers suggested that there is need to harmonize radiological techniques of common X‐ray examinations among diagnostic centers in Nigeria. This would ensure optimal protection of patient during radiological procedure. This study has shown that mathematical method could be adopted in routine medical X‐ray examination for quick determination of dose area product received by patient during the procedure. Furthermore, with the use of appropriate conversion factor, entrance skin dose and effective dose can easily be estimated from calculated dose area product. Future study is aimed at analyzing the impact of increased effective dose due to the combination of the age of X‐ray units used and the technique factors used. In conclusion, the authors wish to recommend that diagnostic X‐ray centers should avoid the use of X‐ray tube greater than 15 years old from the date of manufacture, and the use of high kVp technique must be accompanied with low mAs values for optimal radiation protection of patient.

## ACKNOWLEDGMENTS

The authors wish to express appreciation to the International Atomic Energy Agency, (IAEA) Vienna, Austria and The Abdus Salam International Centre for Theoretical Physics, ICTP, Trieste, Italy, for sponsoring one of the authors in training on Radiation Protection in Diagnostic and interventional radiology, Nairobi, Kenya and Dosimetry in Diagnostic Radiology and its Clinical Implementation, Trieste, Italy. The IAEA fellowship training acquired at the University of Texas, MD Anderson Cancer Center, Houston, Texas, USA is highly appreciated. Also, the cooperation of the patients, radiographers and the management of all the selected centers is greatly appreciated.
